# The value of diffusion kurtosis imaging in assessing mismatch repair gene expression of rectal carcinoma: Preliminary findings

**DOI:** 10.1371/journal.pone.0211461

**Published:** 2019-02-04

**Authors:** Qiang Feng, Hong Yu, Shihang Sun, Zhijun Ma

**Affiliations:** Department of Radiology, Yidu Central Hospital, Weifang Medical University, Qingzhou city, Shandong province, People’s Repubulic of China; Medical University of Vienna, AUSTRIA

## Abstract

**Purpose:**

To determine the correlation between the parameters of MR diffusion kurtosis imaging (MR-DKI) and mismatch repair gene expression (MMR) for rectal carcinomas.

**Materials and methods:**

Data from 80 patients with rectal carcinoma were analyzed in this prospective study. High-resolution T2WI and DKI (b = 0, 800 and 1600 s/mm^2^, respectively) were performed. Mean kurtosis (MK) and mean diffusivity (MD) from DKI were measured. MMR-positive expression and HER-2 expression were classified into two groups. For comparison between different grades, the Mann-Whitney U test, receiver operating characteristic curve, and Spearman’s correlation analysis were used for statistical analyses.

**Results:**

The MK values in identifying positive MMR expressions (MLH1, MSH2, and MSH6) were more reliable than the MD values (r_s_ value: 0.772 vs. 0.448, 0.733 vs. 0.499, and 0.828 vs. 0.633 respectively, *P*<0.01). Receiver operating curve analysis showed that the performances of the MK values were better than those of the MD values (z = 2.835, 2.000, and 2.827, respectively, *P*<0.05), while the performances of the MK and MD-MK values were not statistically significant (z = 0.808, 1.557, and 0.596, respectively, *P*>0.05). Similarly, MK values were better than MD values in identifying *HER2* expression (z = 2.795, *P*<0.05).

**Conclusions:**

MK derived from DKI demonstrated a greater correlation than MD with MMR expression. It also showed better performance in differentiating between high- and low-grade positive MMR expression and *HER2* expression. Thus, DKI may be valuable for the prognoses and evaluation of non-invasive therapies.

## Introduction

Rectal carcinoma is one of the most common malignant tumors [1,2], with an increasing occurrence. The treatment of rectal carcinoma includes surgery and neoadjuvant chemoradiotherapy (CRT). For locally advanced rectal cancer (LARC), CRT is the most commonly treatment, which may reduce the ratio of local recurrence [3] and improve the results of resection. Because of individual differences, the CRT of some patients shows poor or even no treatment response [4], while others show good treatment responses. Early detection and assessment of treatment response before the onset of CRT would be helpful to identify appropriate patients to avoid ineffective and potentially toxic therapy [5].

The expression of mismatch repair (MMR) proteins can be used to predict oncological outcomes and evaluate the prognostic value of overall survival, while the expression of the human epidermal growth factor receptor 2 (HER2) can be used to select the targeted drug [6]. MMR proteins, including MLH1, MSH2, MSH6, and HER2, can only be evaluated by mass biopsy. However, biopsy is invasive and may cause complications. Thus, non-invasive imaging techniques are preferred.

Diffusion kurtosis imaging (DKI) is a non-Gaussian diffusion imaging technique. It provides the kurtosis parameters, including mean kurtosis (MK) and mean diffusivity (MD). DKI is feasible for assessing treatment responses for neoadjuvant CRT in LARC [7] and has been used to assess the histological grades of rectal carcinomas[8]. A previous study did not detect a significant correlation between DKI and MMR.

The purpose of this study was to investigate the correlation between the parameters of DKI and concentrations of the MMR proteins MLH1, MSH2, MSH6, and HER2, and to determine the optimum parameters.

## Materials and methods

### The study population

A total of 130 patients, suspected of having primary rectal carcinoma, were enrolled in the study between December 2016 and August 2018, and underwent an MRI examination in our hospital. Inclusion criteria were as follows: (1) availability of diagnostic-quality preoperative MR images including DKI, (2) absence of any therapy before surgical resection, and (3) histopathologically confirmed rectal adenocarcinoma. A total of 50 patients were excluded for the following reasons: (1) receiving neoadjuvant chemoradiotherapy before DKI (n = 10), (2) having no surgical records in our hospital (n = 13), (3) having other pathological types (n = 12), (4) having DKI images of poor quality because of severe artifacts (n = 11), and (5) having a time interval between the MRI and surgery > 2 weeks (n = 4). Finally, 80 patients with a median age at diagnosis of 56 years (range: 40–70 years), including 35 females and 45 males, were enrolled in this retrospective study. The study was approved by the ethics committee of the Yidu Central Hospital of Weifang Medical University. Written consent was obtained from all participants prior to magnetic resonance imaging (MRI) examinations.

### MRI protocol

All examinations were performed using a 3.0T whole-body scanner (MAGNETOM Skyra; Siemens Medical Solutions, Erlangen, Germany) with a maximum gradient strength of 45 mT/m, and 32 receiver channels. For DKI, fat-saturated coronal EPI sequences were performed in three directions using the Siemens multidirectional diffusion-weighted protocol with b values of 0, 800, and 1600 s/mm^2^.The remaining parameters were as follows: 17 slices; slice thickness, 4 mm with no intersection gap; TR 4000 ms; TE 99 ms; bandwidth, 1898 Hz/pixel; field of view in read direction, 245 mm; field of view in phase direction, 100%; voxel size, 1.9×1.9×4.0 mm^3^; partial Fourier factor, 6/8; number of excitations (NEX), 4; phase encoding direction left to right; the parallel imaging technique, GRAPPA with an accelerator factor 2; and acquisition time was 3 min 18 s. The mass was imaged in an oblique axial orientation slightly tilted perpendicular to the long axis of the rectum. Axial T1-weighted imaging (TR/TE) involved the following: 722/11 ms, 3.0 mm section thickness, 0.3 mm intersection gap, 25 cm field of view (FOV), and 384 × 326 cm matrix. The oblique coronal T2-weighted 2D turbo spin-echo images (TR/TE) involved the following: 4000/99 ms, 3.0 mm section thickness, 0.3 mm intersection gap, 25 cm FOV, and a (384×326) matrix, which was performed before the DKI.

### Immunohistochemistry

Tumor specimens from all 80 patients were obtained. Immunohistochemical staining methods for DNA MMR genes (*hMLH1*, *hMSH2*, and *hMSH6*) and expression of *HER2* were previously described [9,10]. Briefly, a formalin-fixed, paraffin embedded tissue block was used for the tissue microarrays. Multiple sections (4-mm thick) were cut from the tissue and prepared for subsequent immunostaining. The slides were stained with mouse monoclonal antibodies specific for each MMR protein. Negative controls using normal colon epithelium adjacent to the tumor and lymphocytes were performed simultaneously. MMR protein expression levels were classified as negative at < 10% nuclear staining, and positive at > 10% nuclear staining. For positive expression, MMR proteins were divided into low for < 90% (median value of MMR expression) nuclear staining, and high for ≥ 90% nuclear staining [11,12]. For the evaluation of *HER2* expression, ten high-magnification fields were randomly selected and 100 tumor cells were counted using the following criteria: (a) no staining or < 10% of the neoplasms were stained, which was classified as low grade; (b) the positive expression of tumor cell membranes was greater than 10%, which was classified as high grade. If the color was weak and discontinuous, it was defined as (+); if the color of continuous cell membranes encircled the whole cell, it was defined as (++); if the cell membrane of 10% or more of the tumor cells showed a continuous strong positive surface, surrounded by the entire membrane, it was defined as (+ + +). All slides were evaluated in a blinded manner by two experienced gastrointestinal pathologists who had no clinicopathological information.

### Statistical analysis

Statistical evaluation was carried out using SPSS statistical software for Windows (IBM, Armonk, NY, USA). All quantitative variables were first tested for normality analysis using the Kolmogorov-Smirnov test. The MD and MK values of the different MMR proteins between the low and high levels were analyzed by the Mann-Whitney U test, in a manner similar to that of *HER2* expression. The correlations between MD, MK, and percentage of positive MMR protein expression were analyzed by Spearman’s correlation analysis. The diagnostic value of MD, MK, and the combination of MD and MK values (MD-MK) in differentiating levels of MMR proteins and *HER2* expression were analyzed by a receiver operating characteristic (ROC) curve. The corresponding area under the curve (AUC), sensitivity, and specificity were calculated by using a threshold criterion determined as the value that maximized the average of sensitivity and specificity. Differences in diagnostic performance were analyzed by comparing the ROC curves. Inter-observer agreement for the measurements by the two pathologists was analyzed by calculating the interclass correlation coefficient (ICC). A value of P < 0.05 was considered to be statistically significant.

## Results

### DKI of rectal carcinoma

**Fig 1:**
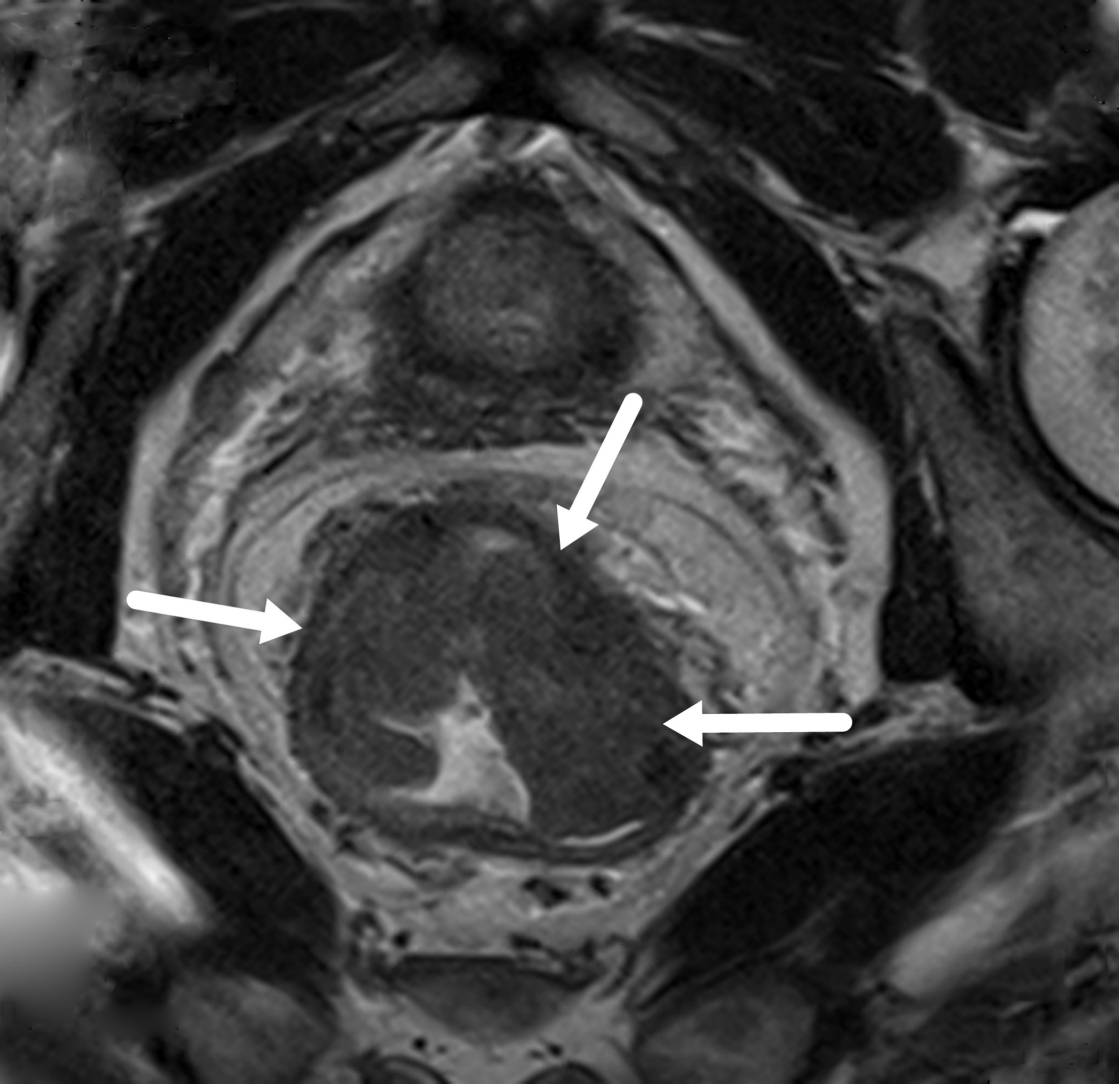
An MR image of a rectal carcinoma. A thickened rectum wall shows slightly higher signals on the T2WI map.

**Fig 2:**
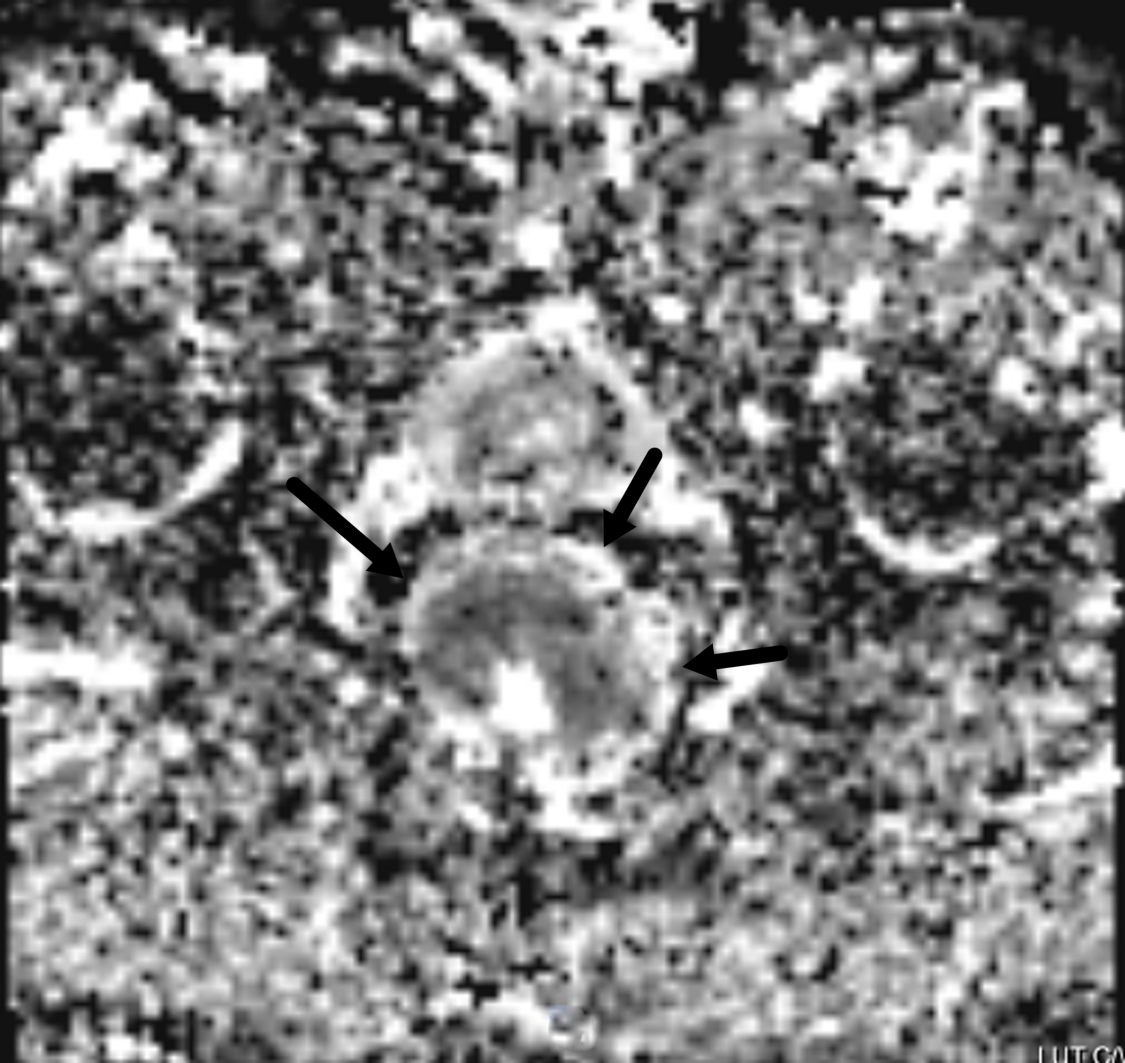
An MR image of a rectal carcinoma. On the MD map, the lesion is the low signal.

**Fig 3:**
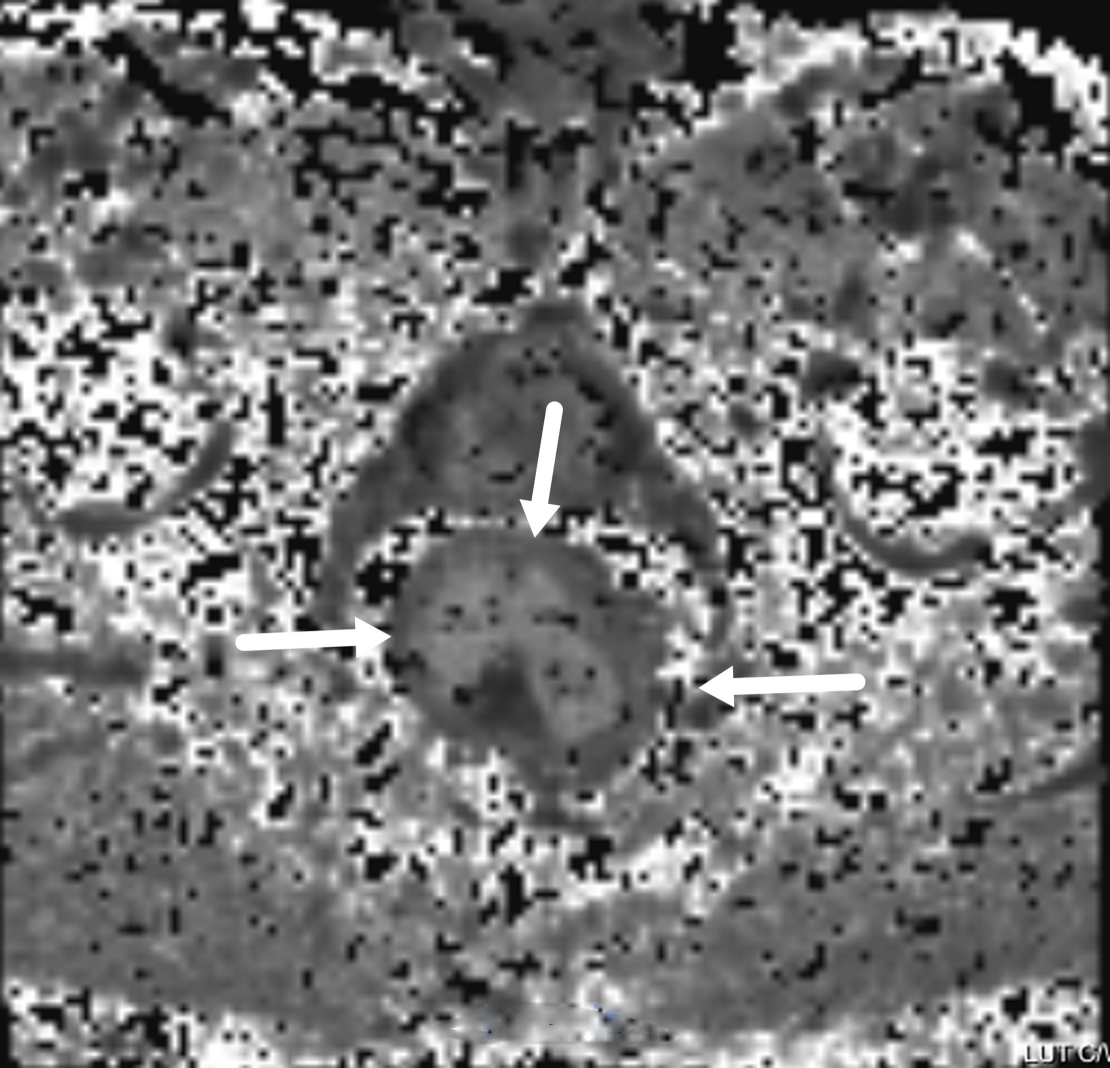
An MR image of a rectal carcinoma. The MK map shows unevenly high signals.

The lesions showed slightly higher signals on T2WI (Fig 1), while relatively homogeneous low signals were shown on MD maps (Fig 2), and the MK maps showed that the tumor signals were comprised of uneven high signals (Fig 3).

### Comparison of MD and MK values between different MMR proteins

**Fig 4:**
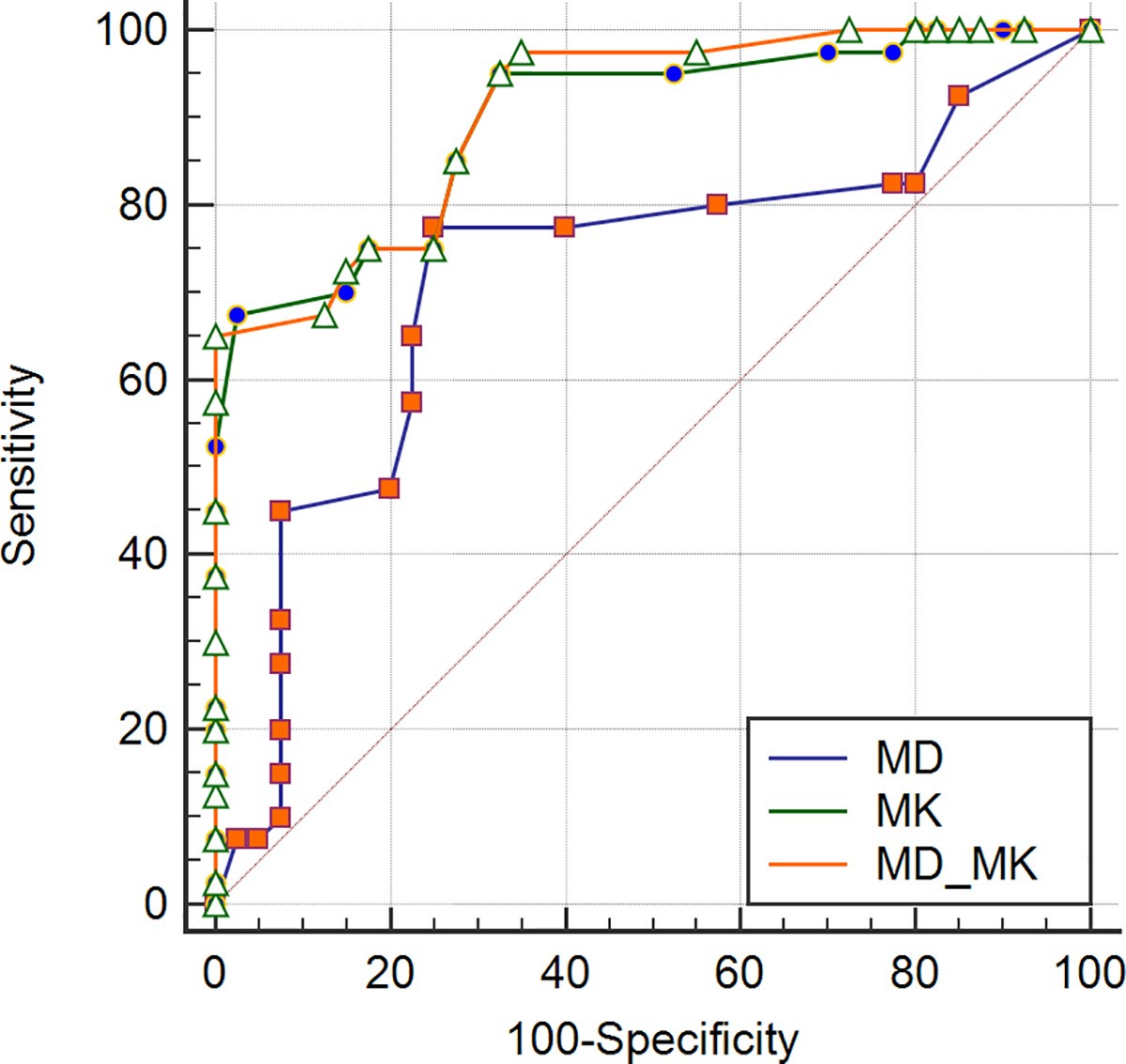
ROC curves displaying the diagnostic performance for MD, MK, and MD-MK in assessing different levels of MLH1.

**Fig 5:**
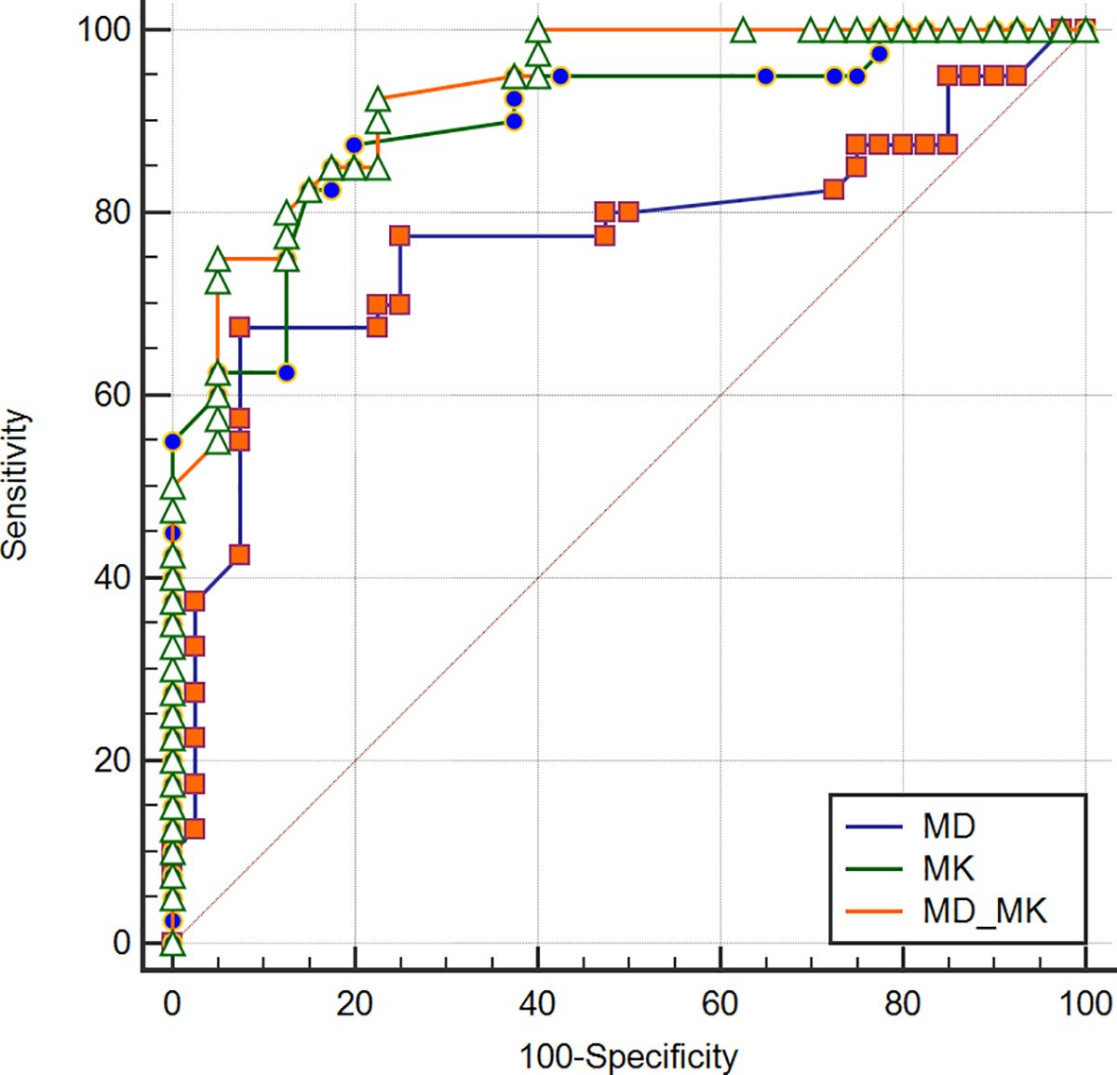
ROC curves displaying the diagnostic performance for MD, MK, and MD-MK in assessing different levels of MSH2.

**Fig 6:**
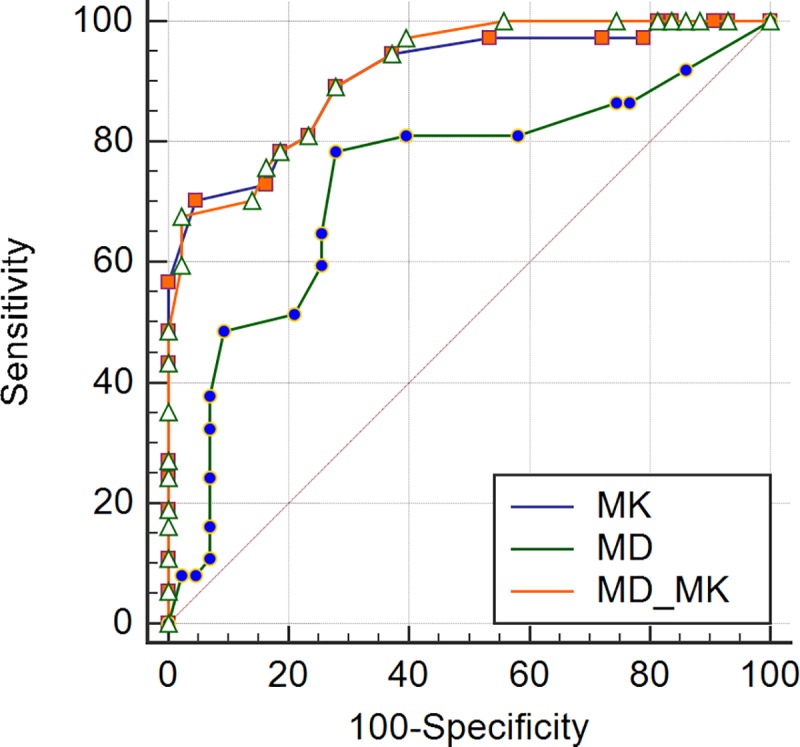
ROC curves displaying the diagnostic performance for MD, MK, and MD-MK in assessing different levels of MSH6.

In our study, MMR protein expression levels were all positive and divided into low and high levels. Overall, the two pathologists agreed closely when measuring MMR positive expression (MLH1, MSH2, and MSH6) in rectal tissues and the ICCs between the values from the two pathologists for measures of each protein were 0.987, 0.952, and 0.901, respectively, so we chose to use the measurements of the first pathologist. For both the MD and the MK values, a statistically significant difference was observed between the low and high expression levels for all MMR proteins (Table 1). Whether using either MD or MK, the correlations with MMR protein expressions were not linear. There was a significant correlation between the percentage of MMR-positive expression and either the MD and MK values. The MK values were more reliable at identifying MMR positive expressions (MLH1, MSH2, and MSH6) than were the MD values (Table 2). ROC analysis data for MD, MK, and MD-MK to assess different levels of MMR protein are summarized in Table 3. For MLH1, MSH2, and MSH6, the performance of the MK value (z = 2.835, 2.000, and 2.827, respectively; *P*<0.05) was superior to that of the MD value, while the performances of the MK value and MD-MK were not statistically significant (z = 0.808, 1.557, and 0.596, respectively; *P*>0.05) (Figs 4–6).

**Table 1:** The comparison of MD and MK between different grades for median (bootstrap-based confidence intervals) levels of MLH1, MSH2, MSH6, and *HER2* expression.

		Low	High	Z	*P*
MLH1	MD	1.32 (1.14–1.40)	1.55 (1.42–1.61)	-3.38	P<0.01
	MK	1.20 (1.14–1.27)	1.11 (1.07–1.15)	-6.12	P<0.01
MSH2	MD	1.32 (1.14–1.54)	1.60 (1.53–1.66)	-4.20	P<0.01
	MK	1.19 (1.14–1.27)	1.08 (1.07–1.11)	-6.14	P<0.01
MSH6	MD	1.31 (1.13–1.40)	1.55 (1.32–1.71)	-3.58	P<0.01
	MK	1.20 (1.16–1.37)	1.11 (1.07–1.14)	-6.26	P<0.01
Her-2	MD	1.32 (1.14–1.61)	1.55 (1.31–1.61)	-1.92	p = 0.06
	MK	1.19 (1.14–1.27)	1.11 (1.07–1.18)	-4.76	P<0.01

Unit of 10^−3^ mm^2^/s for MD.

**Table 2:** Correlations between MD, MK, and MMR protein expression.

Parameter	MLH1	MSH2	MSH6
	r_S_(*P* value)	r_S_(*P* value)	r_S_(*P* value)
MD	0.488 (P<0.01)	0.540 (P<0.01)	0.427 (P<0.01)
MK	-0.772 (P<0.01)	-0.733 (P<0.01)	-0.718 (P<0.01)

MD: mean diffusion; MK: apparent kurtosis; MMR: mismatch repair.

**Table 3:** Receiver operating characteristic analysis of MD, MK, and MD-MK from different MMR proteins.

MMR	Parameter	Threshold	Sensitivity	Specificity	*P*value
MLH1	MD	≤1.399	77.5	75	<0.001
	MK	>1.181	67.5	97.5	<0.001
	MD-MK	≤0.211	65	100	<0.001
MSH2	MD	≤1.399	67.5	92.5	<0.001
	MK	>1.114	87.5	80	<0.001
	MD-MK	≤0.7111	92.5	77.5	<0.001
MSH6	MD	≤1.399	77.78	72.09	<0.001
	MK	>1.181	69.44	95.35	<0.001
	MD-MK	≤0.2555	66.67	97.67	<0.001
HER2	MD	≤1.399	70	67.5	0.056
	MK	>1.130	90	65	<0.001
	MD-MK	≤0.577	90	65	<0.001

Note. Data are 95% confidence intervals. AUC: Area Under Curve MD value are given in mm ^2^ /sec×10^−3^.

### Comparison of MD and MK values at different HER2 expression levels

For HER2 expression, 40 cases (-) and 40 cases (+) were collected in our study and defined as low and high levels, respectively. Regarding both the MD and MK values, there was a statistical difference between the low and high levels of HER2 expression (Table 1).

**Fig 7:**
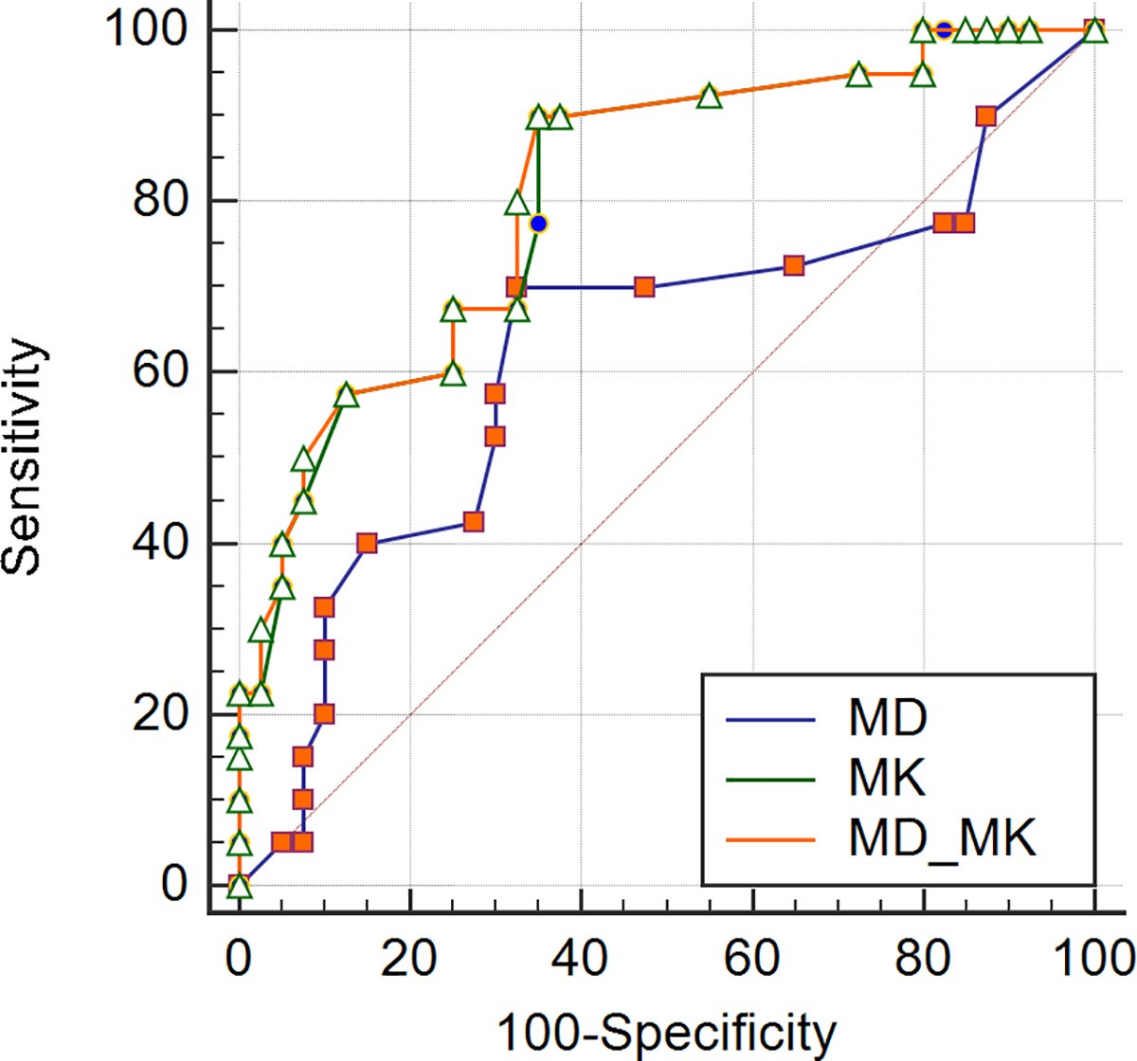
ROC curves displaying the diagnostic performance for MD, MK, and MD-MK in assessing different levels of *HER2*.

ROC analysis data for MD, MK, and MD-MK to assess different levels of HER2 expression are summarized in Table 3. The performance of the MK value was better than the MD value (z = 2.795; *P*<0.05), while the performances of the MK value and MD-MK were not statistically different from one another (z = 1.453; *P*>0.05) (Fig 7).

## Discussion

We assessed the feasibility of a DKI model for assessment of mismatch repair gene expression of rectal carcinoma, to determine whether DKI could be involved in clinical implementation.

A rectal carcinoma at the same stage may follow a different clinical course, so it is essential to identify additional prognostic factors. Novel tissue-based prognostic indicators are necessary for developing new molecular-level therapeutic approaches to rectal cancer [13–15]. It is therefore possible for MMR genes to play both predictive and prognostic roles before and after preoperative therapy in rectal cancer patients [16,17]. DKI showed good performance in differentiating between high- and low- grade MMR positive expression and HER-2 expression.

To date, there have been only a few studies investigating the use of DKI for rectal carcinomas. Yu et al [7] found that DKI had great potential in predicting responses to treatment of rectal cancer, while Zhu et al [8] suggested that DKI could be used successfully to assess histopathological prognostic parameters of rectal carcinoma. To our knowledge, the application of DKI for analysis of rectal carcinoma in the present study was the first to be used for the evaluation of MMR proteins and HER2. Our objectives were to assess the value of DKI in the characterization of rectal carcinoma expressing different levels of MMR proteins and HER2 expressions, and to identify the optimum parameters for DKI.

Moderate positive correlations between MD values and the percentage of MMR-positive expression was observed in this study, but there was a negative correlation between MK and MMR values, which could be explained by defective DNA mismatch repair (dMMR), which failed to correct errors related to genetic abnormalities, leading to tumor formation [18]. The dMMR was identified by the lack of protein expression from the MMR genes, including *MLH1*, *MSH2*, and *MSH6*, which were associated with distinct clinicopathological features, such as a well-differentiated carcinomas, early-stage rectal carcinomas and better prognoses [19]. Compared to poorly differentiated rectal carcinoma, the well-differentiated tumors consisted of a smaller percentage of gland tissue and decreased cell density, with a lower complexity of microstructures. The high MD levels in well-differentiated rectal carcinomas corresponded to high MMR-positive expression. In contrast, MK values were indices of tissue microstructural complexity and heterogeneity [20]. The diffusivity showed a lower performance than kurtosis, a finding which was similar to results from previous studies [8,21]. Diffusivity mainly measures the Gaussian diffusion portions of the diffusion signals from hindered and free diffusion. The results therefore further validated the superiority of the non-Gaussian distribution model.

*HER2* expression is related to local invasion depth, adjacent structural invasion, and distant metastasis of colorectal cancer, and can be used as an independent factor to evaluate prognoses of colorectal cancer patients [6]. The high expression of *HER2* showed a lower MD value and a higher MK value than did low *HER2* expression in this study. A possible reason may have been that high expression of the HER2 receptor induced cells to proliferate excessively and inhibited cell apoptosis, which led to tumor formation and more aggressive growth. At the molecular level, the water motion may have been restricted, and the heterogeneity might have been the main reason for the greater kurtosis.

This study had some limitations. Firstly, the sample size was low, and negative expression of the MMR protein and *HER2* expression with (++) and (+++) were not recorded in this study. Secondly, this was a retrospective study, and the results may not have represented the features of the general population. A prospective study with more patients is needed in the future.

In conclusion, DKI identified MMR protein expression associated with rectal carcinoma using the values of MD and MK. MD correlated positively with MMR protein expression, but MK showed an opposite relationship. Thus, DKI may be valuable for the prognoses and the evaluation of non-invasive therapies.

## Supporting information

S1 File This is the raw data. (XLS)
